# Impact of Leading Line Composition on Visual Cognition: An Eye-Tracking Study

**DOI:** 10.16910/jemr.17.5.2

**Published:** 2024-12-13

**Authors:** Hsien-Chih Chuang, Han-Yi Tseng, Chia-Yun Chiang

**Affiliations:** Chinese Culture University, Taiwan; Shih Hsin University, Taiwan

**Keywords:** photography, composition, leading lines, eye tracking, visual guidance

## Abstract

Leading lines, a fundamental composition technique in photography, are crucial to guiding the viewer’s visual attention. Leading line composition is an effective visual strategy for influencing viewers’ cognitive processes. However, in-depth research on the impact of leading line composition on cognitive psychology is lacking. This study investigated the cognitive effects of leading line composition on perception and behavior. The eye movement behaviors of 34 participants while they viewed photographic works with leading lines were monitored through eye-tracking experiments. Additionally, subjective assessments were conducted to collect the participants’ perceptions of the images in terms of aesthetics, complexity, and directional sense. The results revealed that leading lines significantly influenced the participants’ attention to key elements of the work, particularly when prominent subject elements were present. This led to greater engagement, longer viewing times, and enhanced ratings on aesthetics and directional sense. These findings suggest that skilled photographers can employ leading lines to guide the viewer’s gaze and create visually compelling and aesthetically pleasing works. This research offers specific compositional strategies for photography applications and underscores the importance of leading lines and subject elements in enhancing visual impact and artistic expression.

## Introduction

Advances in digital technology and the widespread use of social media have amplified photography’s social and cultural influence. In the modern era, the ubiquity of cameras has made photography an essential medium for expression and communication. The rapid dissemination of photographic works influences people’s values and lifestyle, underscoring the critical role of composition in photography. Peterson (8) emphasized that the effective arrangement of elements in an image is more crucial than the image’s content itself if a photograph is to be compelling. Carroll (30) considered composition a key factor in imposing order on seemingly chaotic scenes and creating visually impactful images. Photographers employ clever compositional techniques to design appropriate scenes, transforming spatial and depth perceptions into flat visual effects. Different compositional methods—such as symmetry, the rule of thirds, framing, and focal composition (69)—have distinct characteristics and visual effects. Of these, leading lines, symmetry, and the rule of thirds are particularly noteworthy (30). Symmetry and balance have significant perceptual effects on composition (41). Effective composition involves the artist systematically arranging compositional elements to guide the viewer’s gaze, creating a unified and harmonious whole that enables the viewer to focus on the most prominent subject and experience the emotions conveyed by the photographer, thereby leaving a lasting impression on the viewer. Even without professional artistic training, viewers can still achieve a clear and compelling visual experience (6, 7; 30, 39).

As noted by Tokuyuki Ishida (64) and Freeman (47, 49), pointing is a highly effective technique for directing a viewer’s gaze due to humans’ natural tendency to follow lines. Visual guidance plays a crucial role in enhancing visual flow and focusing attention. Leading lines—a widely used compositional strategy in photography, painting, and design—offer a powerful means of visual guidance. In photography, elements such as roads, rivers, and buildings can serve as leading lines. These lines, whether prominent or subtle, guide the viewer’s gaze. Freeman (49) noted that skillful use of leading lines by photographers can guide the viewer’s gaze, indicate direction, and provide pathways, resulting in a clear and natural viewing experience through focus on the core of the image. In particular, photographs that incorporate leading lines and subject elements tend to be more visually appealing than those not doing so; the lines and elements highlight the theme and create engaging and memorable photographic works.

Photographic theory has developed a set of rules for good composition - the rule of thirds, golden ratio, triangular composition and central perspective. etc. are all about creating images of beauty, and research evidence shows how that affects beauty. Freeman (49, 47) underscored the importance of balance and harmony in compositions. Understanding how viewers perceive images is crucial for photographers. Although compositional techniques influence viewing behavior, a gap exists in knowledge regarding the principles governing the movement of visual attention. Few studies have investigated how viewers observe photographs, and empirical research on these laws remains scarce (38). Albert (23) highlighted the importance of understanding the impact of leading in a composition on the viewer’s gaze. Eye-tracking technology offers a valuable tool for measuring gaze, recording sequences of viewing events, and revealing cognitive processes through gaze trajectories. With this technology, researchers can understand how viewers observe and are attracted to key elements of images (70; 18; 49; 54). Empirical support for compositional theories in photography psychology research is limited, and relatively few studies have combined art with cognitive science, but eye-tracking analysis techniques have become widely adopted in psychological research.

This study investigated the subjectivity of leading line composition in photography and its impact on visual attention. By employing eye-tracking experiments to obtain gaze trajectories, fixation points, heat maps, and subjective evaluations, this study elucidated the effects of leading line composition techniques on viewers’ gaze behavior and attention focus distribution as well as the influence of subject elements on visual effects. This research contributes to a deeper understanding of the psychological mechanisms and patterns involved in appreciating photographic works. Additionally, it can help enhance the visual effectiveness and appeal of photography, providing substantial theoretical and empirical support for photographers and artists. Ultimately, this study can advance the development of visual art theory and improve image creation.

The research hypotheses were as follows:

Hypothesis 1: When a leading line composition includes a prominent subject element, the subject's presence will significantly attract viewers' attention, enhancing their focus on key positions within the image. Therefore, leading line compositions with a subject are expected to result in longer fixation durations.

Hypothesis 2: Leading line compositions with prominent subject elements will significantly enhance viewers' experiences of aesthetics and sense of direction, leading to higher subjective evaluations of the image.

## Literature Review

### Composition: An Invisible Power Center

Peter Ward (52) emphasized the pivotal role of composition in photography and described it as a vital yet often overlooked aspect. Renowned photographer Edward Weston famously stated, “Composition is the strongest way of seeing.” Composition involves skillful arrangement and organization of visual elements within a flat space, establishing meaningful relationships between them to create an organized whole. This is essential for producing outstanding artistic works (25; 30; 32; 33; 13; 44; 71). The primary objective of composition is to create visual harmony and unity, resulting in a clear and organized aesthetic. By carefully designing and manipulating visual elements and their relationships within a work, composition effectively communicates themes and emotions. In the visual arts, composition strongly influences visual perception, guiding the viewer’s gaze and enhancing a work’s visual appeal and message delivery (4; 56). Effective photographic composition can offer fresh visual interest and guide the viewer’s gaze (52). By skillfully integrating subjects and visual elements, photographers can organize an image neatly, achieving visual harmony and balance, as balance is a fundamental design principle in the highest artistic practices (41). This, in turn, means that the image’s message is effectively conveyed, its subject is emphasized, and it has depth and clarity. Compositional methods help focus the viewer’s gaze on key parts of the image, guiding their vision so they can easily understand the emotion or story expressed (21; 52). Barr (21) highlighted that most people initially focus on the main subject of a photograph before gradually expanding their attention to the overall content. Alternatively, the viewer can be guided by lines and thereby move through the photograph in a structured manner.

### Role of the Main Subject Element in Composition

**Figure 1.**
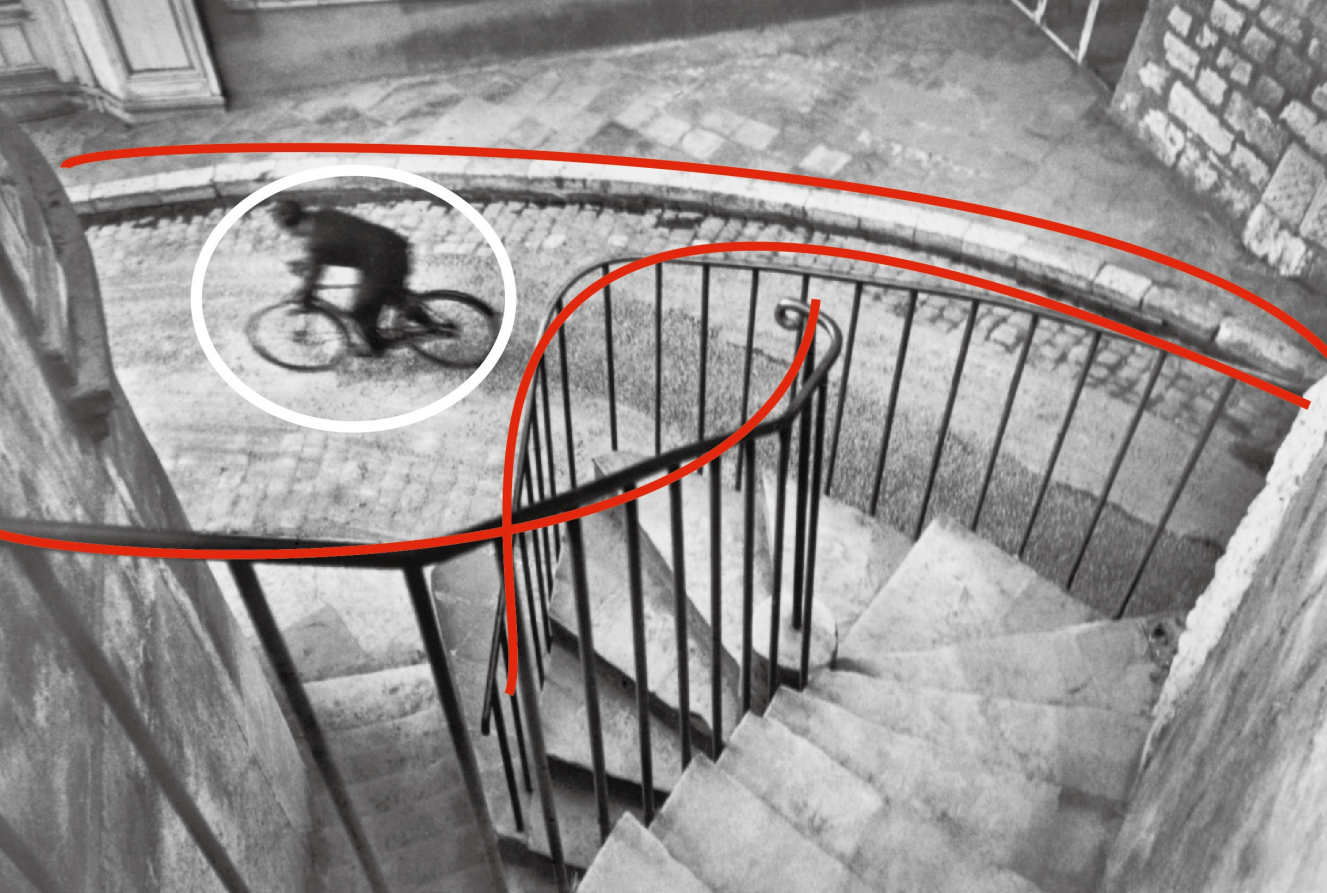
Henri Cartier-Bresson, The Var department. Hyères, 1932

**Figure 2.**
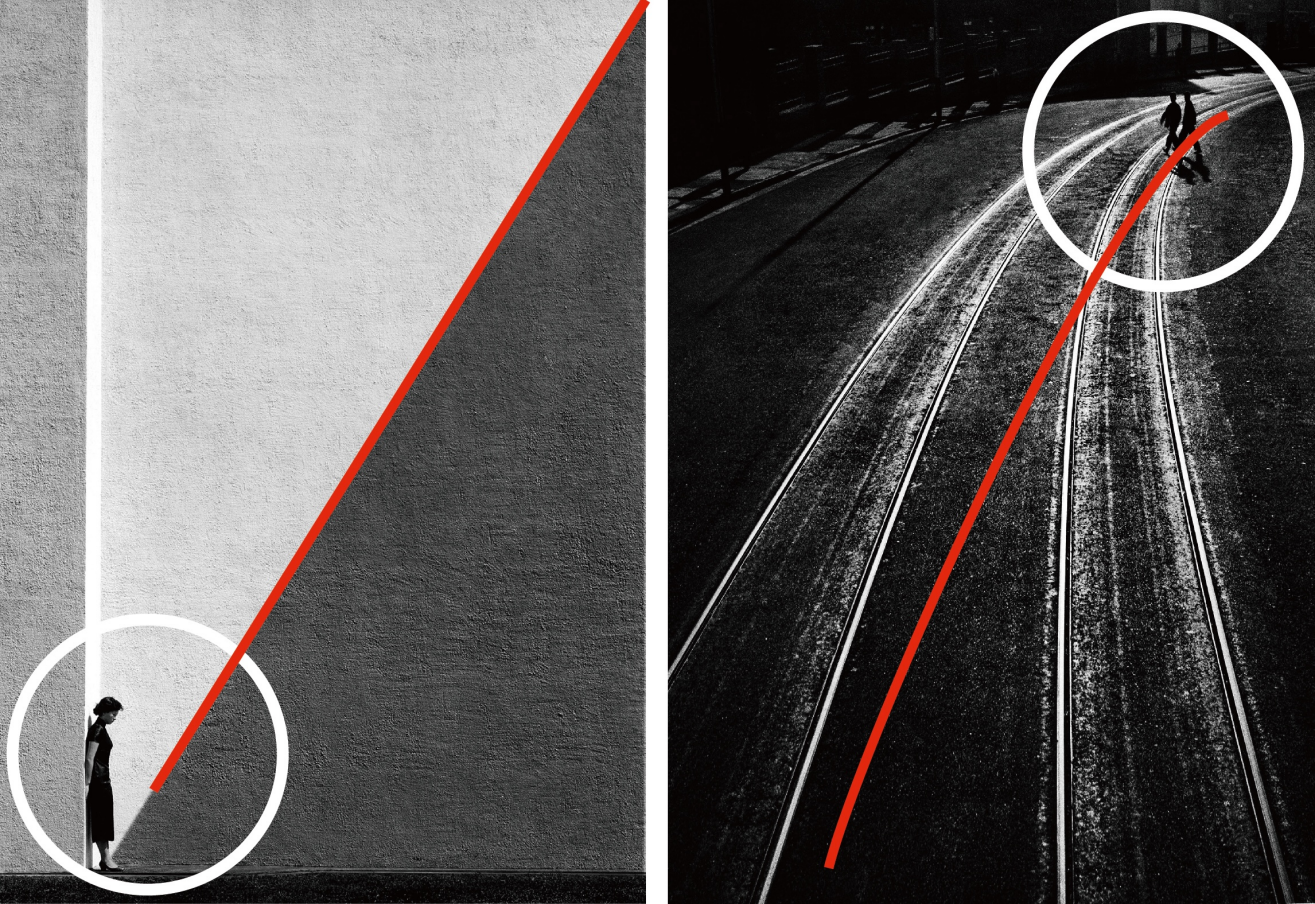
Fan Ho, Approaching Shadow, 1954

Leading line composition is a common and effective technique that uses faintly visible or explicit lines as visual elements to guide the flow of the viewer's attention to the main subject which can produce a coordinated and harmonious visual experience (8; 48; 23; 20; 30; 14; 52). For example, in Henri Cartier-Bresson's 1932 classic, he used railings as guiding lines to guide the audience's sight in a curved and moving manner (as shown in Figure 1). Fan Ho, a well-known Hong Kong photographer, is good at using lines to guide vision, focus the audience's attention on important locations, and create extended visual effects (as shown in Figure 2).

Albert (23) stated that works without subjects or characters will appear vacant, bland and disturbing, and disorienting to the viewer (48; 52; 49; 52; 30; Carroll, 2017). On the other hand, Peterson (8) pointed out that a composition with a subject can add interest and enhance the strength of the composition. He used photography to compare the difference between with and without a subject, and found that photos employing guided compositions with a subject, are more attractive those without a subject. The viewer's attention will be significantly focused on the subject, which becomes the center of interest of the picture. Therefore, the use of themes and center of interest to convey the creator's emotions, arouse the audience's resonance and psychological feelings, and capture the audience's attention and focus, is very important (23).

### Eye Movement

The eyes serve as windows to perception and cognition and provide valuable insights into psychological processes (5). Eye-tracking technology uses eye-trackers to record and analyze eye movement. According to Rayner (54), research on eye movements has focused on three basic types: fixations, saccades, and smooth pursuit. These eye movements are fundamental to understanding how the human visual system processes information (1; 53; 66).

The rapid advances in modern eye-tracking technology have enabled the precise measurement of eye movements in various fields and contexts. Valuable insights have been obtained into visual information processing and perception by using real-time visual focus and dwell time data as scientific evidence. Early eye movement research focused on vision and psychology, the fields most directly concerned with visual perception. Currently, eye-tracking technology is being employed in a wide range of applications, including medicine (35), human–computer interfaces (16), music (15), visual search (63), design, and film (10; 59; 61). Locher, P. J. (40) pointed out that eye tracking technology is a very effective tool that can reveal the perceptual and cognitive processes of the aesthetic experience of visual art. By overlaying the viewer’s eye movement data with the images they view, a scanpath can be drawn to reveal which elements capture the viewer’s interest and trigger exploratory behavior.

In art research, Buswell (9) demonstrated that studying eye movements can reveal where the viewer focuses their attention in images (12; 68). He was the first to conduct experiments linking eye movements with image viewing to explore the changes in individuals’ eye movement behavior and perception that occur when they view photographs and artworks. The study by Buswell revealed that initial viewing involves shorter fixations, whereas longer viewing times result in longer fixations and a lower frequency of saccades. Moreover, viewers tend to fixate on spatial locations similar to those found in the image, although the sequence of fixations varies among individuals. Molnar (50) analyzed differences in saccade amplitude and fixation duration between classical and Baroque paintings and discovered larger saccade amplitudes and lower saccade speeds for classical works and denser eye movements and smaller amplitudes for Baroque works. These findings suggest an association between eye movement patterns and the perception of image style.

Studies on photographic creation and compositional forms are particularly notable in the field of photography and cognition. Chuang et al., (11) employed eye tracking and Gestalt theory to analyze visual cognition processes and revealed that Gestalt images significantly influenced fixations, the gaze distribution, and subjective assessments of aesthetic appeal and complexity. Moreover, closed composition images were perceived as more straightforward and accessible and resulted in fewer fixations and saccades, longer fixations, and a more concentrated gaze. Eye movement research provides crucial information on attention, enabling researchers to gain deep insights into fixation locations and attention distributions. These data, in turn, enable a better understanding of the underlying cognitive psychological processes (2; Chuang, 2017; Cumming, 1978; 17; 28; 29; 34; 36; 43; 45; 53; 54; 57; 62; 66).

### Dependent variables

1. Total number of fixations: Fixation refers to the eye focusing on a specific target while remaining relatively still, with alignment of the target and fovea to optimize visual acuity (67). During fixation, the brain is processing information (19). Although the eyes may appear stationary during fixation, they exhibit slight drifts, tremors, and microsaccades (37). Fixations typically last between 200 and 300 ms, but this duration can vary depending on the type of stimulus. For example, fixations are generally shorter when reading text than when viewing images (24; 46). Yarbus (70) demonstrated that when viewing a painting, observers frequently return to the most important parts of the image. Consequently, analyzing the distribution of fixation points on an image can reveal the areas on which people most often focus. The common variables relating to fixation are the number of fixations (65). Goldberg and Kotval (22) and Henderson and Ferreira (27) noted that a higher number of fixations often indicates processing difficulty, and that materials that are harder to view tend to exhibit higher number of fixations.

2. Total fixation duration: it is the number of times the eyes fixate on a specific area, and the fixation duration, which is the duration of each fixation, measured in milliseconds, and may reflect the task being internally processed, processing, personal preferences, or the complexity of the external stimulus. Richer information and more attractive subjects typically result in longer fixations (3; Chuang & Tseng, 2023; 26; 34; 42; 51; Salvucci & Anderson, 1998; Shimonishi & Kawashima, 2020; Tseng & Chuang, 2024).

3. Number of saccades: The count of the occurrence of saccades which are rapid eye movements in which attention is shifted from one fixation point to another, facilitating the acquisition and exploration of visual information.

4. Saccade duration: The length of time for a single saccade, the duration for the eye to move from one fixation point to the next. Saccades are a common form of eye movement, and the eye moves at speeds up to 800°/s in saccades (72). Compared with fixations, saccades are shorter, typically ranging from 20 to 35 ms (53).

5. Heat Map: A visualization tool that typically uses varying color intensities to represent the density or intensity of data. The heat map converts the recording of the entire stimuli viewing process into an intuitive visual image to reveal areas of concentration and fixation durations. Multiple recordings enhance understanding of sightline consensus and attention distribution (Bojko, 2013 ; 65)

6.Aesthetics Evaluation: It refers to the viewer's assessment of the aesthetic qualities and emotional response to photographic works and a comprehensive judgment of their appeal. Berlyne (1974) found that when viewers develop a preference for a visual work, it triggers a range of aesthetic emotions and synesthetic experiences, including interest and pleasure. These aesthetic responses reflect the individual's immediate perception of the work and reveal their aesthetic preferences within different contexts and cultural frameworks.

7. Complexity: This refers to the subjective perception of the complexity in the structure, content, or design of photographic works and the cognitive load it induces. It is influenced by factors such as the number of elements, information density, and the diversity of visual stimuli and is related to the viewer's perception, motivation, and aesthetics (Attneave, 1957; Chipman, 1977; Chipman & Mendelson, 1979; Michailidou, 2005; Michailidou et al., 2008; 54; 65). Leder et al., (38) suggest moderate image complexity enhances aesthetic appeal, but excessive complexity may reduce attractiveness.

8. Sense of direction: In photography this refers to arranging elements such as composition, lines, lighting, and colors to guide the viewer's ga ze, creating a clear visual direction and dynamic movement within the artwork. This sense of direction enhances the photo's depth and three-dimensionality, increasing the image's tension and emotional expression. Directing the viewer's attention toward the main subject makes the theme more distinct. However, the sense of direction should serve the expression of the theme without overwhelming it, ensuring that the core idea of the work remains prominent.

## Methodology

### Experimental Design

The composition of photographic works is crucial for shaping visual impressions and conveying emotions. Leading line composition, a vital visual guiding technique, employs explicit or subtle lines to considerably influence the viewer’s viewing path and perceptual experience. Eye-tracking technology was employed in this study to investigate the application of leading line composition in photographic works and its impact on visual cognition.

The primary objective of this study was to analyze the influence of different photographic creation techniques on viewing patterns. Specifically, this study explored the influence of leading line composition on eye movement information and gaze distribution. In the experimental design, leading lines and subject elements in photographic works were manipulated to analyze their key effects on viewer behavior. Through systematic variation of these elements, a deeper understanding of how subject elements guide gaze flow and influence attention could be obtained.

**Figure 3.**
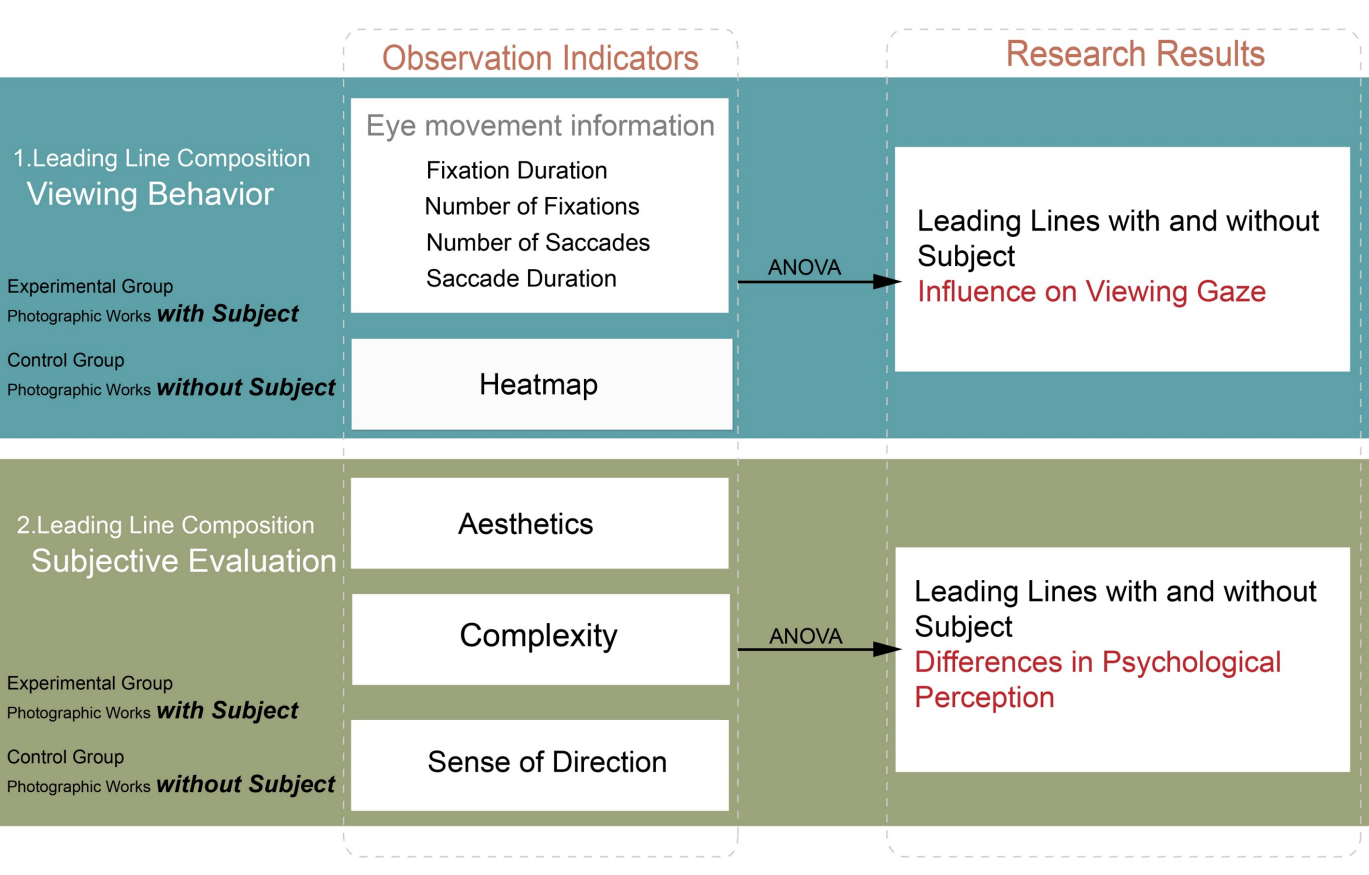
Research Framework

The focus of the study was to investigate how leading line composition and subject elements influence viewers’ gaze trajectories, gaze distribution, and fixations as well as their effect on an image’s visual appeal. Subjective evaluations were made by 34 participants and combined with eye movement data to explore the influence of leading lines and subject elements on participants’ emotional responses and aesthetic judgments. The research framework is presented in Figure 3. This study provides insights into the effects of leading line composition techniques in photographic works and empirical support for photographers to enhance their work’s visual impact and artistic value.

### Participants

Thirty-four volunteers were recruited—16 male and 18 female participants aged between 18 and 25 years. All participants were native Chinese speakers and university students. The participants were selected through a paid recruitment process. The experimental design involved a completely randomized presentation of stimulus materials and a within-subject design. This ensured that each participant was exposed to all experimental conditions, thereby ensuring the comprehensiveness and reliability of the eye movement data.

### Experimental Stimuli

**Figure 4.**
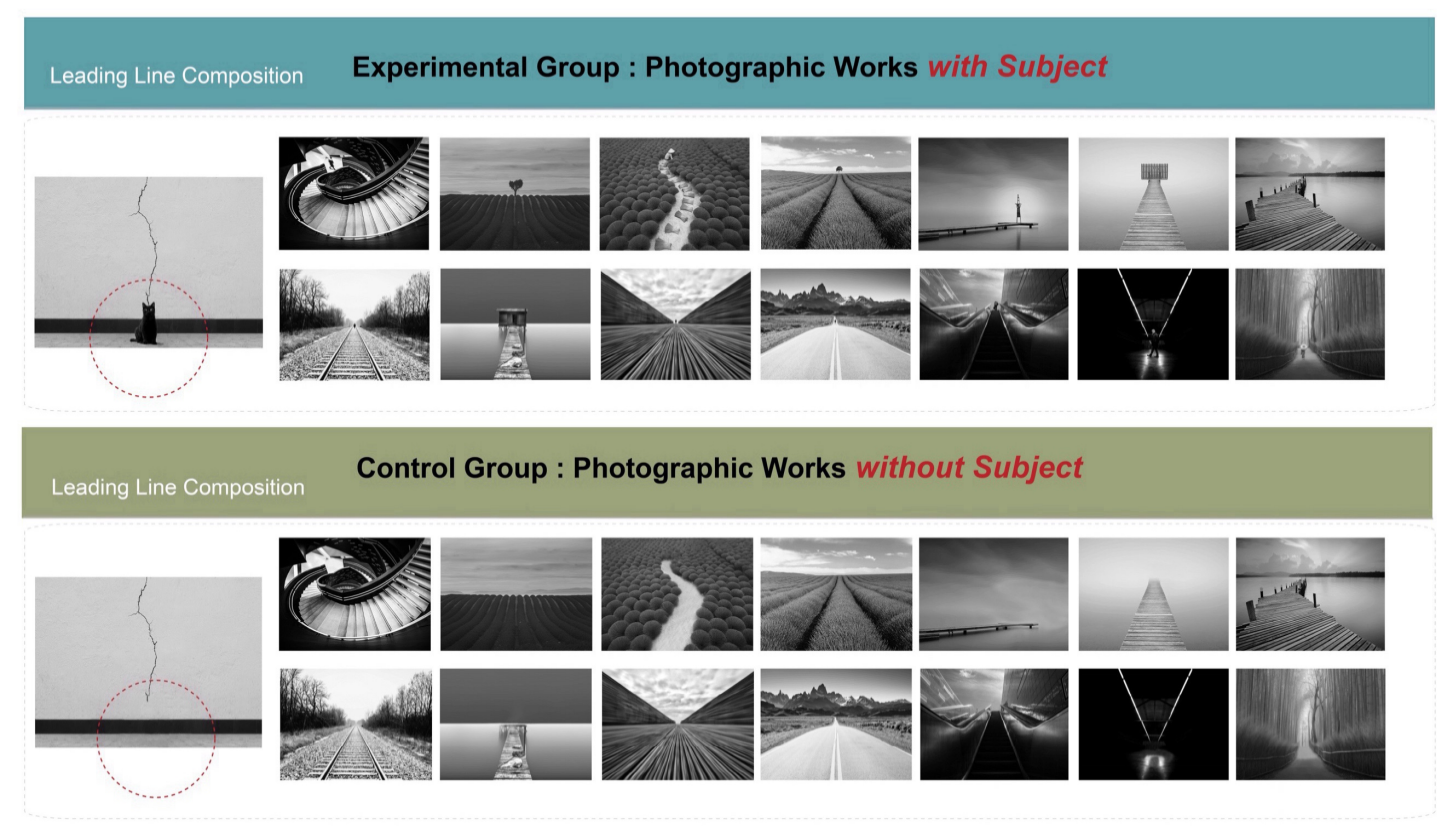
Experimental Stimul

The experimental photographs were evaluated and selected by three experts with over 15 years of photography experience. By using a Likert scale, they chose 15 photographic works that had a composition with a leading line. Each photograph with a subject was then processed to remove the subject elements, resulting in 15 photographs without subjects. The total number of black-and-white experimental photographs was thus 30. All photographs had resolution of 1,920 × 1,440 pixels (Figure 4).

For the experimental manipulation, a controlled experimental design was employed to systematically vary the presence of leading lines and subject elements within the photographic works. Eye movement behavior was recorded using an eye tracker. The independent variable in this study was the presence or absence of subject elements in a composition with a leading line (experimental group: photographs with subject elements; control group: photographs without subject elements). The dependent variables were eye movement information (e.g., total number of fixations, total fixation duration, number of saccades, and saccade duration) and subjective evaluations (e.g., participants’ ratings on a photograph’s aesthetics, complexity, and sense of direction).

### Experimental Procedure

Prior to participating in the experiment, the participant was instructed to sit comfortably in a controlled environment, maintaining a distance of approximately 60 cm from a 21-inch LCD monitor with their eyes aligned horizontally with the center of the monitor. The Tobii Pro Nano eye tracker was employed to record eye movement at 60 Hz. The tracker underwent nine-point calibration to ensure the stability and accuracy of the data it produced. Before commencing the formal experiment, the participant completed a practice session and read the experimental instructions to familiarize themselves with the procedure and equipment.

**Figure 5.**
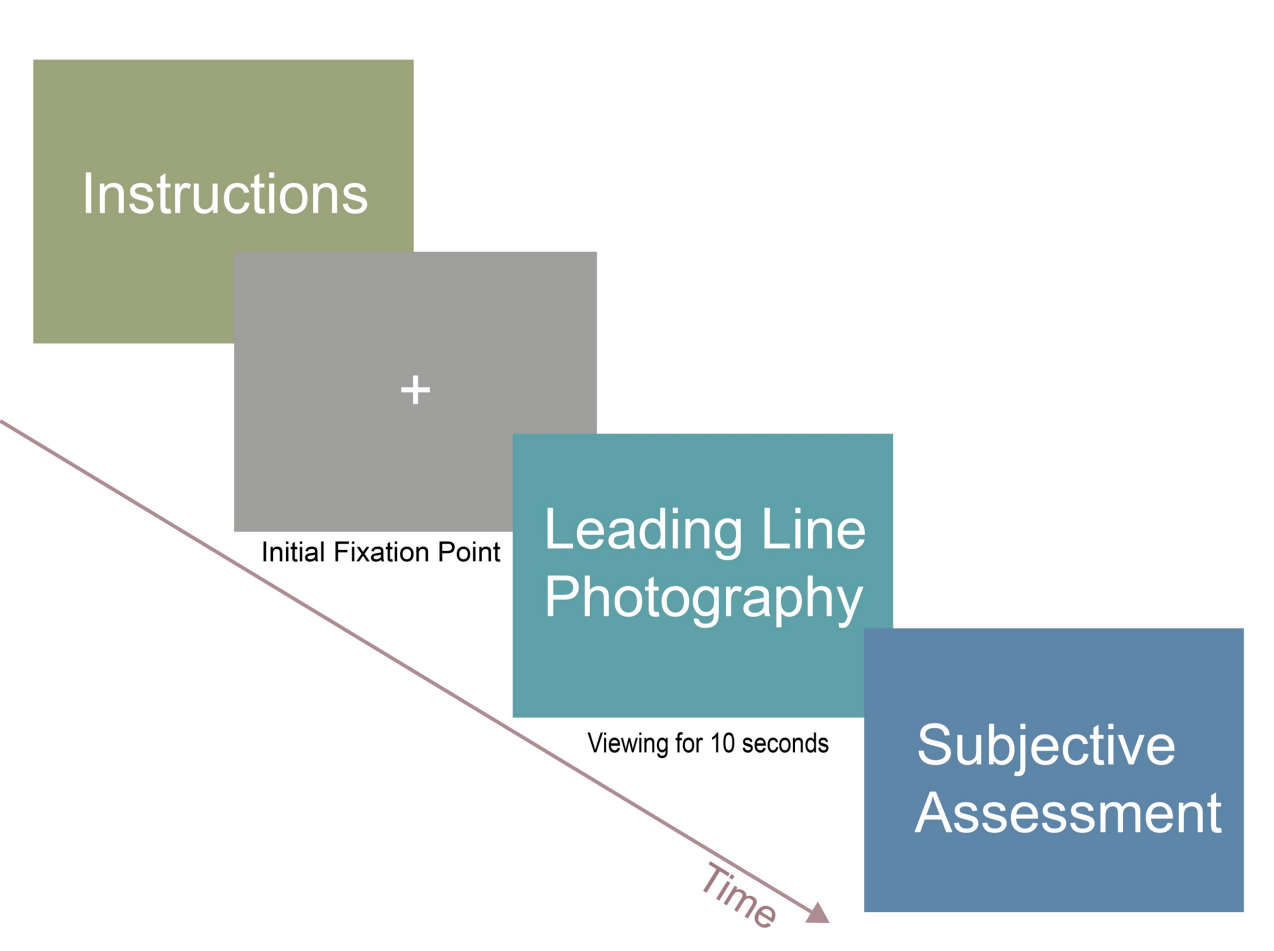
Experimental Procedure

During the experiment, the participant was required to view 30 randomly presented photographic works, with each image displayed for 10 s, resulting in 30 trials. Following the viewing session, the participant provided subjective evaluations of each photograph in terms of their complexity, aesthetics, and sense of direction on a 5-point Likert Scale, with 1 indicating *very simple* or *very poor* and 5 indicating *very complex* or *very good*. The entire experimental procedure lasted approximately 10 min. The detailed experimental process is illustrated in Figure 5.

## Results

### Impact of the Presence of Subjects on Viewer Gaze

**Figure 6.**
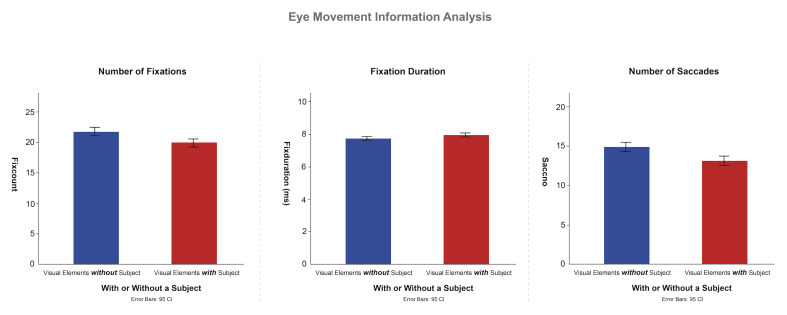
Eye Movement Information for Photographs with and without Subjects

This study investigated the effect of the presence of subjects in leading line compositions on the distribution of the participants’ gaze, with a specific focus on the number and duration of fixations. Two-way analysis of variance (ANOVA) was conducted to assess differences in viewing behavior due to the presence versus absence of a subject (Figure 6). The results indicated significant main effects of subject presence on the number of fixations [*F*(1, 29) = 31.657, *p*< .001] and fixation duration [F(1, 29) = 6.248, *p*< .05]. These findings suggest that the presence of subjects in photographic works significantly influences overall viewing behavior. Specifically, photographs with subjects led to the participants having fewer fixations but significantly longer viewing times. The presence of subjects is thus confirmed to attract the viewer’s interest, leading to a prolonged focus on the main object for a deeper observation and understanding of the work.

Two-way ANOVA was conducted to investigate the impact of the presence of subjects in leading line compositions on saccadic movements. Saccadic behavior, characterized by rapid eye movements between different focal points, is commonly used to assess the structural complexity and information density of content. The analysis revealed a significant main effect of subject presence on the number of saccades [*F*(1, 29) = 24.437, *p*< .001], indicating that the presence of a subject significantly influences overall gaze behavior. Specifically, photographs with a subject had a clear focal point that attracted the participants’ interest, resulting in fewer saccades. By contrast, photographs that lacked a subject or a clear visual focus led to the participants searching for information, resulting in more saccades. However, significant main effect on saccade duration was also discovered [*F*(1, 29) = 6.937, *p*< .05], indicating that the presence of a subject presence did significantly affect the duration of saccades.

**Figure 7.**
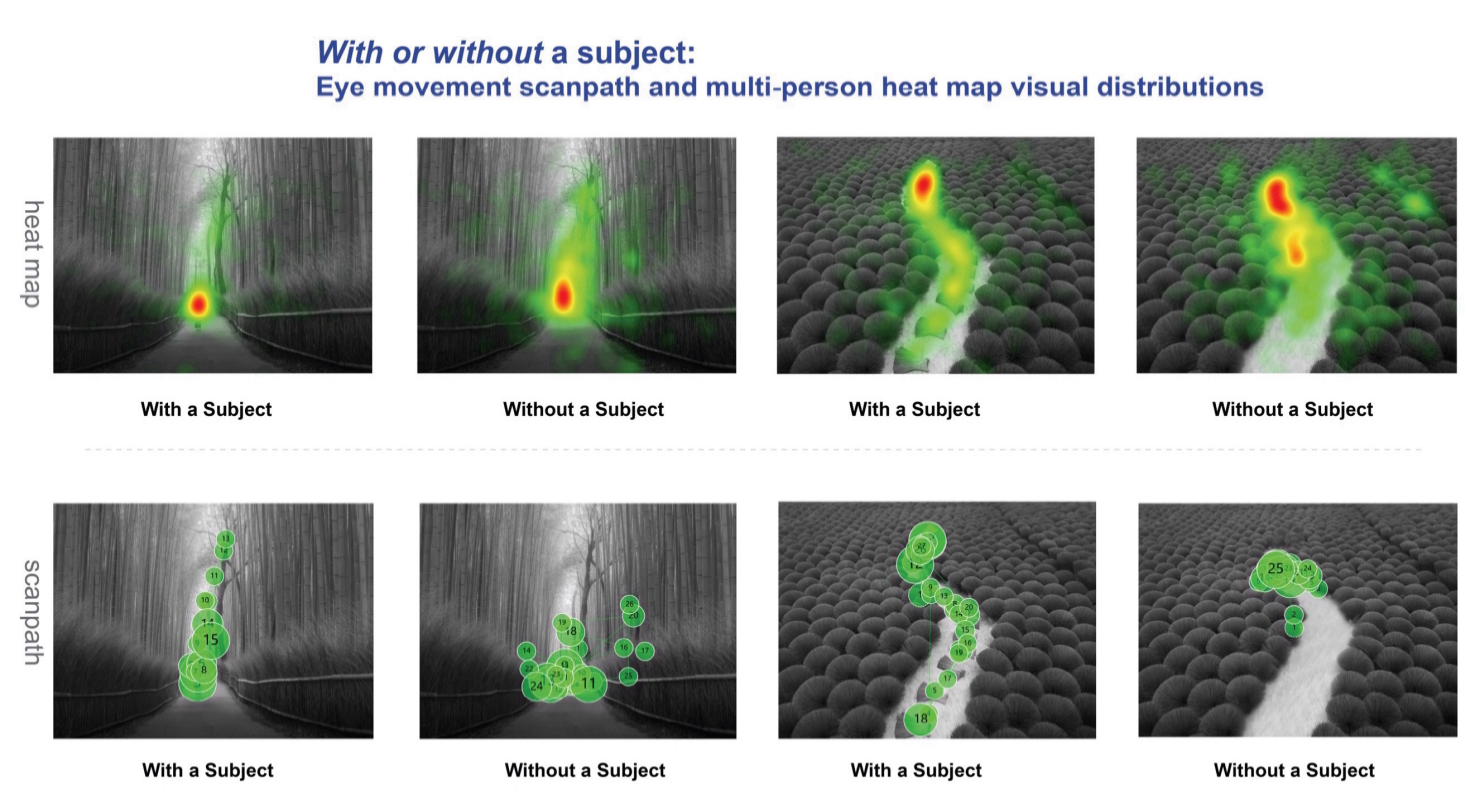
Gaze Trajectories and Multiviewer Heatmaps for Photographs with Leading Line Compositions

Figure 7 presents this study’s qualitative multiviewer heatmaps and scan paths, which have previously been found to illustrate the distribution of viewers’ attention and fixation locations (59; 53). When viewers examine photographs with a leading line composition, their gaze naturally follows the leading line, demonstrating the key role of leading lines in regulating visual attention and gaze direction. Leading lines effectively focus the viewer’s attention on primary visual elements or subjects, enhancing their concentration and understanding of the work.

### Impact of the Presence of Subjects on Subjective Ratings

**Figure 8.**
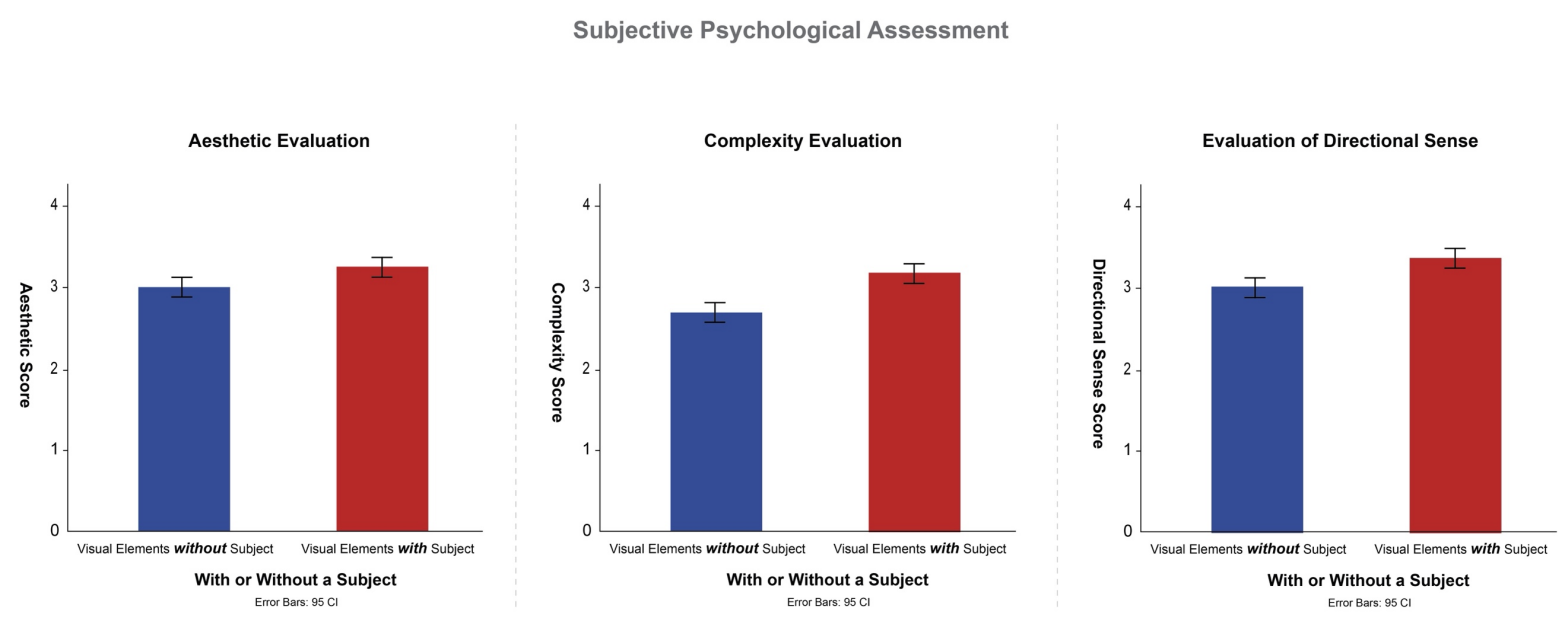
Effect of Clear Subject Elements on Aesthetic and Directional Ratings

In the subjective assessment, the participants rated the photographs in terms of their aesthetics, complexity, and sense of direction. The results revealed that the presence of subject elements significantly enhanced the participants’ aesthetics and sense of direction ratings (Figure 8). Specifically, the analysis related to aesthetics indicated that photographs with subjects received significantly higher ratings than did those without subjects [*F*(1, 33) = 4.631, *p*< .05]. Additionally, the complexity analysis revealed that photographs with subjects were perceived as more complex by the participants [*F*(1, 33) = 16.134, *p*< .001]. The evaluation of sense of direction revealed that photographs with subjects were more effective in guiding the direction of the participants’ gaze [*F*(1, 33) = 10.565, *p*< .01], , Indicating that subject elements can effectively direct visual flow. These findings suggest that incorporating prominent subject elements into photographic compositions can improve the work’s aesthetics and sense of direction. However, an excessive number of subject elements may result in the work appearing overly complex. Therefore, photographers should carefully balance the arrangement of leading lines and subjects to enhance their work’s appeal and visual impact.

## Conclusions

This study integrated objective eye-tracking data with subjective evaluations to investigate the effects of leading line compositions and subject elements in photography on visual cognition and psychological responses. The findings underscore the pivotal role of the skillful use of leading lines and subject arrangement in photography. By effectively employing lines and layout techniques, photographers can direct the viewer’s attention to specific locations within the artwork, highlight visual focal points, and enhance the work’s aesthetic appeal and overall visual experience, thereby achieving more effective visual communication (47, 49).

Uchiike and Fukuif (31) indicated that a well-developed sense of direction can guide the viewers’ gaze, making the image appear more natural, which aligns with the views of Carroll (30), Zakia and Page (71), and Ward (52). Additionally, the presence of subject elements was discovered to have a substantial impact on the participants’ gaze. When photographs included a clear subject, they attracted more interest from the participants, considerably reduced saccadic movements, and increased fixation duration, supporting Hypothesis 1, while enhancing ratings for aesthetics and sense of direction, thereby supporting Hypothesis 2. Relevant studies have indicated that photographs with subjects have greater interest, higher compositional power, and more effectively capture the viewer’s attention, consistent with the conclusions of Ward (52), and Peterson (8), and the results from this study, as illustrated in Table 1.

**Table 1.**
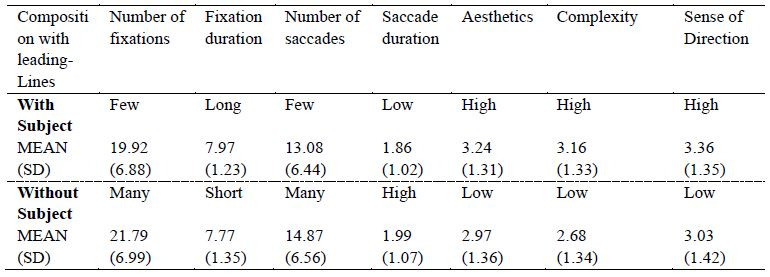
summary of the results

Research on the relationship between aesthetic preferences and image structure (Beaumont, 1985; Freimuth & Wapner, 1979; Mead & McLaughlin, 1992; 41) indicates that cueing directionality and arranging focal points with subject elements can enhance perceived balance in composition, thereby improving the aesthetic appeal and visual attractiveness of the work (41). Additionally, regarding complexity, this study also found that as visual elements become more complex, the perceived complexity of works with subjects increases. Leder et al., (38) noted that moderate image complexity can enhance aesthetic appeal, but excessive complexity may reduce attractiveness. Therefore, appropriately arranging elements in photographic compositions can effectively enhance aesthetics, and maintaining moderate complexity makes the work more appealing. This also explains the positive correlation between complexity and aesthetics in this study.

This study underscores the pivotal role of leading line composition and subject elements in photographic creation and reveals the relationship between composition and visual cognition processes. The findings offer concrete guidance for photographers, highlighting the importance of the judicious use of leading lines and subject elements to effectively direct the viewer’s attention, enhance visual cognitive effects, and foster a deeper understanding and appreciation of the artwork. In terms of research limitations, subjective complexity and aesthetic evaluations can vary depending on personal preference, cultural background, and circumstance. In addition to manipulating the subject's presence or absence, this study will more rigorously design a control group with or without obvious leading lines in conducting future experiments. Future research could explore the application and visual psychological impacts of leading line compositions in various cultural and media contexts, such as posters in print media and dynamic advertising videos. Such investigations would contribute to a better understanding of how different media formats influence viewers’ attention allocation, providing additional empirical support and design recommendations.

### Ethics and Conflict of Interest

The authors declare that the contents of the article are in agreement with the ethics described at http://biblio.unibe.ch/portale/elibrary/BOP/jemr/ethics.html and that the authors have no conflicts of interest regarding the publication of this paper.

### Acknowledgement

The authors thank Dr. Da-Lun Tang of Tamkang University for his assistance with the consultation on statistical analysis.
